# Walking Erectly

**Published:** 1869-05

**Authors:** 


### WALKING ERECTLY .

NOT only adds to manliness of appearance, but develops the chest and promotes the general health in a high degree, because the lungs being relieved of the pressure made by having the head downward and bending the chest in, admit the air freely and fully down to the very bottom of the lungs.

If an effort of the mind is made to throw the shoulders back, a feeling of tiredness and awkardness is soon experienced, or it is forgotten. The use of braces to hold up the body is necessarily pernicious; for there can be no brace which does not press upon some part of the person more than is natural, hence cannot fail to impede injuriously the circulation of that part. But were there none of these objections, the brace would soon adapt itself to the bodily position, like a hat or shoe or new garment, and would cease to be a brace.

To maintain an erect position or recover it when lost, in a manner which is at once natural, easy and efficient, it is only necessary to walk habitually with the eyes fixed on an object ahead, a little higher than your own, the eve of a house, the top of a man's hat, or simply keep your chin a very little above a horizontal line, or, it will answer to walk with your hands behind you ; if either of these things is done, the necessary, easy, and legitimate effect is to relieve the chest from pressure, the air gets in more easily, developes it more fully, and permeates the lungs more extensively, causing a more perfect purification of the blood, imparting higher health, more color to the cheek, and compelling a throwing out of the toes. To derive the highest benefit from walking, hold up the head, keep the mouth closed, and move briskly.

